# Monitoring changes in malaria epidemiology and effectiveness of interventions in Ethiopia and Uganda: Beyond Garki Project baseline survey

**DOI:** 10.1186/s12936-015-0852-7

**Published:** 2015-09-04

**Authors:** Tarekegn A. Abeku, Michelle E. H. Helinski, Matthew J. Kirby, Takele Kefyalew, Tessema Awano, Esey Batisso, Gezahegn Tesfaye, James Ssekitooleko, Sarala Nicholas, Laura Erdmanis, Angela Nalwoga, Chris Bass, Stephen Cose, Ashenafi Assefa, Zelalem Kebede, Tedila Habte, Vincent Katamba, Anthony Nuwa, Stella Bakeera-Ssali, Sarah C. Akiror, Irene Kyomuhagi, Agonafer Tekalegne, Godfrey Magumba, Sylvia R. Meek

**Affiliations:** Malaria Consortium, London, UK; Malaria Consortium, Addis Ababa, Ethiopia; Malaria Consortium, Kampala, Uganda; Rothamsted Research, Harpenden, UK; Medical Research Council/Uganda Virus Research Institute, Uganda Research Unit on AIDS, Entebbe, Uganda; South Nations, Nationalities and Peoples Regional Health Bureaux, Hawassa, Ethiopia; London School of Hygiene & Tropical Medicine, London, UK; Ethiopian Public Health Institute, Addis Ababa, Ethiopia; National Malaria Control Programme, Ministry of Health, Kampala, Uganda

**Keywords:** Malaria, *Plasmodium falciparum*, *Plasmodium vivax*, *Anopheles gambiae*, *Anopheles arabiensis*, *Anopheles funestus*, Vector control, Epidemiology, Undernutrition

## Abstract

**Background:**

Scale-up of malaria interventions seems to have contributed to a decline in the disease but other factors may also have had some role. Understanding changes in transmission and determinant factors will help to adapt control strategies accordingly.

**Methods:**

Four sites in Ethiopia and Uganda were set up to monitor epidemiological changes and effectiveness of interventions over time. Here, results of a survey during the peak transmission season of 2012 are reported, which will be used as baseline for subsequent surveys and may support adaptation of control strategies. Data on malariometric and entomological variables, socio-economic status (SES) and control coverage were collected.

**Results:**

Malaria prevalence varied from 1.4 % in Guba (Ethiopia) to 9.9 % in Butemba (Uganda). The most dominant species was *Plasmodium vivax* in Ethiopia and *Plasmodium falciparum* in Uganda. The majority of human-vector contact occurred indoors in Uganda, ranging from 83 % (*Anopheles funestus* sensu lato) to 93 % (*Anopheles gambiae* s.l.), which is an important factor for the effectiveness of insecticide-treated nets (ITNs) or indoor residual spraying (IRS). High *kdr*-L1014S (resistance genotype) frequency was observed in *A. gambiae* sensu stricto in Uganda. Too few mosquitoes were collected in Ethiopia, so it was not possible to assess vector habits and insecticide resistance levels. ITN ownership did not vary by SES and 56–98 % and 68–78 % of households owned at least one ITN in Ethiopia and Uganda, respectively. In Uganda, 7 % of nets were purchased by households, but the nets were untreated. In three of the four sites, 69–76 % of people with access to ITNs used them. IRS coverage ranged from 84 to 96 % in the three sprayed sites. Half of febrile children in Uganda and three-quarters in Ethiopia for whom treatment was sought received diagnostic tests. High levels of child undernutrition were detected in both countries carrying important implications on child development. In Uganda, 7–8 % of pregnant women took the recommended minimum three doses of intermittent preventive treatment.

**Conclusion:**

Malaria epidemiology seems to be changing compared to earlier published data, and it is essential to have more data to understand how much of the changes are attributable to interventions and other factors. Regular monitoring will help to better interpret changes, identify determinants, modify strategies and improve targeting to address transmission heterogeneity.

## Background

The malaria burden has declined worldwide in the past decade. Malaria mortality rates decreased by 47 % globally and by 54 % in the WHO African Region between 2000 and 2013 [1]. During the same period, prevalence of infection in children aged 2–10 years decreased from 26 to 14 %—a 48 % decline [1]. Factors that are believed to have had a significant on this trend impact include the scale-up of key vector control interventions, availability of rapid diagnostic tests (RDTs) and effective treatment with artemisinin-based combination therapy (ACT) [2, 3]. However, the trend has not been uniform. In some areas with high baseline transmission and/or where high coverage levels have not been achieved, the malaria burden has not declined [4–7].

The changing epidemiology of malaria requires adaptation of interventions to address shifts in geographical, behavioural and demographic risk characteristics, especially as transmission declines and becomes more clustered [8]. A deeper understanding of possible determinants of change is critically important. Local knowledge of the burden and features of the disease will be important to adapt interventions and maintain cost-effectiveness and equity. Features that need to be monitored include: changes in vector habits and insecticide resistance, parasite infection patterns and drug resistance, climatic, socio-economic and demographic changes, gaps or issues in implemented interventions, and effectiveness and relevance of some control strategies.

Furthermore, a good surveillance system is essential to identify most at-risk populations and geographical areas and to assess trends and impact of interventions [9]. In addition to surveillance and monitoring of empirical data, appropriate mathematical models are useful to understand the malaria transmission dynamics. Examining various scenarios, including the extent to which a set of interventions can reduce malaria to low levels, could help to use resources optimally.

Detailed epidemiological studies have been carried out with the aim of understanding the likelihood of interruption of malaria transmission in Africa. One of the best examples of such studies was the epidemiological research undertaken in the Garki Project during 1969–1976 in a lowland rural Sudan savanna in northern Nigeria [10]. The goal was to test the effects of indoor residual spraying (IRS) and mass drug administration and to develop and test a mathematical model of transmission. Although potent interventions were applied, interruption of transmission was not achieved. Part of this failure was attributed to non-uniform exposure to sprayed surfaces due to at least partial genetically determined outdoor resting populations of *Anopheles gambiae* s.l. However, the model constructed by the project proved useful for planning malaria control interventions. More recently, a malaria model was developed, which has been proposed to be used in elimination scenario planning [11, 12]. Models could be used to extrapolate realistic predictions in larger geographical areas for selective control planning and evaluation of effectiveness of interventions in bringing down transmission to a low level. Data from the present project could be used to validate such models and to stratify areas for optimum impact within available resources. The project which is the subject of this paper was named ‘Beyond Garki’ to recognize the contribution of the Garki Project to the understanding of malaria epidemiology in Africa.

The project is led by Malaria Consortium and implemented in collaboration with the Ethiopian Public Health Institute and Ministries of Health in Ethiopia and Uganda, alongside Regional/District Health Offices in the study sites. Here, the project is described and data on several variables presented, including malaria epidemiology, vector behaviour and insecticide resistance, demographic and socio-economic factors, treatment-seeking behaviour and coverage of interventions in the study sites from a baseline survey carried out in October and November of 2012. Three more rounds of surveys have been carried out up to November 2014. The detailed results of these surveys and other data in comparison with the baseline survey will be published elsewhere.

## Methods

### Study sites

A ‘study site’ in the context of the project is defined as a ‘health centre and the catchment population in selected villages around it’. Two study sites were selected per country in Ethiopia and Uganda, representing different epidemiological settings in rural environments (Table 1).

**Table 1:** Beyond Garki study sites in Uganda and Ethiopia

Country	Region	District	Study site	Coordinates of health centre: latitude, longitude	Average altitude of study site (m)
Uganda	Northern Region	Apac	Aduku	1°59′33.51″N, 32°43′8.26″E	1051
	Central Region	Kyankwanzi	Butemba	1°8′33.86″N, 31°36′8.79″E	1107
Ethiopia	SNNP Region	Boloso Sore	Hembecho	7°8′59.08″N, 37°39′42.05″E	1702
	SNNP Region	Halaba Special	Guba	7^o^17′6.88″N, 38^o^13′1.09″E	1878

*SNNP* Southern Nations, Nationalities and Peoples

The selection of the study sites was based on the need to represent different epidemiological (transmission) settings, geographical location and accessibility, and availability of adequate baseline morbidity data. The four study sites represented settings ranging from low seasonal transmission in the Ethiopia sites to high perennial transmission in the Uganda sites. Only villages in close proximity to the health centres were selected, covering a radius of approximately 2–6 kms to reduce potential bias in the analysis of treatment-seeking and use of services by the study population.

#### Ethiopia

Most areas below 2000 m above sea level are considered malarious in Ethiopia. An estimated 60 % of the population live in areas at risk of malaria transmission [13]. Both *Plasmodium falciparum* and *Plasmodium vivax* are common. The Malaria Indicator Survey (MIS) during October-December 2011 showed that nationally the prevalence of malaria was 1.3 % in areas below 2000 m; 77 % of the positive slides were *P. falciparum* infections [14]. There is marked seasonality in transmission and geographic variation in intensity. Many areas are epidemic-prone. *Anopheles arabiensis* is the main vector species [15]. *Anopheles pharoensis*, *Anopheles funestus* and *Anopheles nili* are considered secondary vectors. Resistance of the main vector against DDT and pyrethroids is widespread in the country [16].

Ethiopia’s organized malaria control began in 1959 when the Malaria Eradication Service was established a year after a major epidemic claimed an estimated 150,000 lives [17]. A blanket DDT spraying campaign was used until the early 1970s, when the eradication strategy was abandoned and replaced with a control programme [18]. The programme, based on selective spraying and treatment of cases, continued until the mid-1990s after which the specialized service was gradually integrated into the general health services. There has been a substantial increase in coverage of key interventions in the country. More than 64 million long-lasting insecticidal nets (LLINs) were distributed through mass campaigns between 2005 and 2014 [13]. IRS is also implemented in many areas. Through the expansion of basic health services, mainly health posts, diagnostic and treatment services have increased over the years.

#### Uganda

Malaria is highly endemic in approximately 95 % of the country where 90 % of the population live. The MIS in November and December 2009 reported that 42 % of children under the age of five tested positive for malaria with microscopic diagnosis [19]. *Plasmodium falciparum* is responsible for 99 % of malaria cases. The disease accounts for 25–40 % outpatient visits and nearly half of inpatient paediatric deaths [20]. The main malaria vectors are *Anopheles gambiae* s.s., *A. arabiensis* and *A. funestus* [19, 20].

Although IRS was implemented in limited sites as part of the WHO pilot programme between 1959 and 1963, the operation was not scaled up [21]. Treatment of cases remained the only malaria control measure for many years. The Malaria Control Unit was established in 1995 and grew into the National Malaria Control Programme. The main preventive interventions in Uganda are LLINs, IRS in selected districts, and intermittent preventive treatment in pregnancy (IPTp). Uganda has scaled up effective case management and in some regions village health teams (VHTs) were trained to test and treat common childhood illnesses including malaria through Integrated Community Case Management (ICCM). In 2009, 47 % of households owned at least one insecticide-treated net (ITN) compared to 16 % in 2006 [19, 22]; this increased to 60 % in 2011 [23]. These combined efforts are believed to have resulted in reduced transmission in many areas [24].

Up to 10 districts in northern Uganda have been sprayed in the past 6–7 years within the IRS programme supported by the US Government’s Presidential Malaria Initiative (PMI) [20]. Starting from 2014, more northern and eastern districts were added while the operation ended in others (including Apac, the district containing the study site Aduku) due to a decline in transmission. A large reduction in malaria prevalence was observed in children living in sprayed areas compared to those living in unsprayed areas [25]. Meanwhile, more than 21 million LLINs were distributed in a nation-wide mass campaign during 2012–2014.

### Study components

Repeat cross-sectional surveys were conducted in the selected sites (of which only results from the first study are presented here as the baseline data). The study also included longitudinal collection of meteorological and morbidity data at health facilities. The main components include: household surveys, malariometric and serological surveys, entomological surveys, health facility-based morbidity studies, and climatic studies. For the household surveys, the required sample size was estimated for each site by assuming 5 and 50 % baseline prevalence in children below 10 years in Ethiopia and Uganda, respectively.

#### Household surveys

All households in villages around each health centre (within radius of 2–6 kms) were enumerated and included in the sampling frame. A simple random sample of 571 and 234 households were selected in each site in Ethiopia and Uganda, respectively. The sample sizes were determined separately for the two countries based on expected malaria prevalence rates, household sizes and a 10 % non-response rate using appropriate statistical procedures, and sample sizes for each site were calculated independently assuming simple random sampling. The household surveys included interviews with household heads and women aged 15–49 years of age using handheld devices (smartphones with Pendragon Forms 5.1 or tablets with Open Data Kit). Data were collected on variables indicating socio-economic status (SES), prevention methods, knowledge about malaria, ITN ownership and use, as well as number of children born to interviewed women who were alive and dead, febrile illness in children, treatment sought and protection against malaria during pregnancy.

#### Malariometric surveys

Each member of the sampled households (except infants under 6 months) was given a subject card and asked to visit a malariometric testing site within the village to obtain anthropometric measurements and collect blood samples. Bodyweight, temperature, height and mid-upper arm circumference were measured for children under five. Thick and thin blood films for microscopy, dry blood spots for serology and blood samples for haemoglobin measurement using the HemoCue machine (Hb 301, Ängelholm, Sweden) were obtained for all subjects. RDTs (CareStart™ pf-HRP2/pan-pLDH by Access Bio USA in Ethiopia and SD Bioline in Uganda) were used to test subjects with body temperature 37.5 °C and above or history of fever in the previous 48 h. Individuals with fever or history of fever were tested by RDT for the purpose of providing anti-malarial treatment. Treatment was provided at the field site according to national guidelines for mild and moderate anaemia (using ferrous sulphate) and uncomplicated malaria cases (using artemether–lumefantrine for *P. falciparum* and chloroquine for *P. vivax*), while severe cases were referred to the site’s health centre.

Slides were stained with Giemsa and examined by two independent microscopists for presence/absence of asexual parasites and gametocytes and species identification. In the case of discrepant results, a third microscopist examined the slides for a final verification.

Serological analysis of dry blood spots from the Uganda sites was carried out to determine antibody responses to assess malaria transmission intensity over an extended period of time. The antibody response of individuals against merozoite surface protein-1_19_ (MSP-1_19_) was determined using an enzyme-linked immunosorbent assay (ELISA). Serum obtained from the dried blood spots on filter papers was analysed at the Medical Research Council (MRC) Laboratory in Uganda for total IgG antibodies using *P. falciparum* antigen MSP-1_19_ (CTK Biotech, USA, cat. No. A3003) following previously described methods [26, 27].

#### Entomological surveys

*Anopheles* mosquitoes were sampled to determine species composition, densities, behaviour and insecticide resistance using light trap collection, exit trap collection, room search, pyrethrum spray catch and human landing catch (HLC) in 12 houses per site selected using simple random sampling from the sampling frame for the household survey. Mosquitoes were identified using morphological features and individually packed in microtubes for molecular analysis, which were carried out at Rothamsted Research in the UK [28]. Genomic DNA was extracted using the Livak method. *A. gambiae* s.l. samples were analysed to determine whether they were *A. gambiae* s.s. or *A. arabiensis* [29, 30]. *Anopheles gambiae* s.l. samples were analysed mainly for knock down resistance (*kdr*) mutations (but also for mutation in the *ace*-*1* gene which encodes the acetylcholinesterase enzyme although not reported here) [31].

#### Other study components

Other components of the study not reported in the present paper include: use of automatic weather stations (BWS200 automatic weather station, Campbell Scientific, Stellenbosch, South Africa) installed in all sites to record hourly meteorological data, compilation of outpatient morbidity data for every suspected or confirmed malaria patient seen at the health facility in each site, and mathematical modelling of transmission.

### Ethical considerations

Ethical clearance was obtained from the appropriate review boards (Uganda: UNCST 1348; Ethiopia: 3-10/819/05). In addition, written consent was obtained from respondents for interviews, for all subjects that participated in malariometric surveys, and from household heads for entomology sentinel houses.

### Data entry and analysis

EpiData v3.1 (The EpiData Association, Odense, Denmark) was used for data entry where necessary. Stata versions 12 and 13 (StataCorp LP, College Station, TX, USA) and Microsoft Excel (Microsoft Corporation) were used for data analysis.

#### Household survey data

Chi squared tests were used where appropriate to assess significant differences between groups of interest, taking into account clustering at household level. Principal components analysis (PCA) was used to calculate wealth index for each household, computed separately for each country.

#### Malaria infection rates

Infection prevalence data were analysed in relation to potential household or individual risk factors such as coverage and use of prevention methods, housing conditions, demographic factors and socio-economic status.

#### Undernutrition

Undernutrition was studied using the anthropometric data for children under five. A Stata program file ZSCORE06 developed by Jef Leroy (Boston College Department of Economics) was used to calculate anthropometric z-scores using the 2006 WHO Child Growth Standards [32].

#### Anaemia

Anaemia was classified as mild, moderate or severe based on the concentrations of haemoglobin (Hb) as follows; (a) mild anaemia: for non-pregnant women, Hb 10.0–11.9 g/dl; for pregnant women and children under 5, Hb 10.0–10.9 g/dl; for men: Hb 10.0–12.9 g/dl; (b) moderate anaemia: Hb 7.0–9.9 g/dl; c) severe anaemia: Hb <7.0 g/dl [33].

#### Serological studies

Optical density (OD) values were analysed in Microsoft Excel using a macro file provided by C. Drakeley, London School of Hygiene and Tropical Medicine (LSHTM). Normalized OD values were used for data analysis using a Stata procedure provided by C. Drakeley (LSHTM). A cut-off value of 0.177 was used to determine seropositive samples. Age seroprevelance curves were generated using methods described by Corran et al. [34]. Data for children below 2 years was excluded to avoid potential bias caused by maternal antibodies [35].

#### Under-5 mortality rates

A variant of the Brass indirect method [36] was used to calculate under-five mortality rate (U5MR) using the summary birth history dataset provided by women of child-bearing age which included age of mother, total number of live births and total number of deaths [37]. Mortality rates were not calculated for the 3 years period before the survey date (2009–2012), namely data related to women aged 15–19, because of the selection effect where women from lower socioeconomic classes tend to start childbearing early and their children face above average mortality risks [38]; and because random errors are larger for estimates based on the reports of young women, since they have fewer children ever born.

#### Entomological data

Various entomological parameters were estimated including species compositions, and indoor resting and biting habits. Human biting rates (i.e., the number of bites per person per night) were calculated taking into account the number of collectors working simultaneously, the number of collection nights, and the assumed night-time behaviour of the local human populations. It was assumed that an average villager in each of the sites spends 1 h on average outdoors between 18:00 h and 22:00 h, and all villagers are indoors after 22:00 h.

## Results

A total of 1521 households with complete records were included in the analysis of this first survey from the four sites in Ethiopia (average 540 per site) and Uganda (average 221 per site). Household response rates were 94.6 and 94.4 % in Ethiopia and Uganda, respectively. In total, 8079 people were registered. Nearly all the registered household members were usual residents, ranging from 97.2 % in Aduku (Apac district, Uganda) to 100 % in Guba (Halaba Special district, Ethiopia). Females constituted 51.6–53.2 % of the populations in the study sites.

Blood samples for microscopy and serology and body temperature measurements were obtained for 66 % of registered household members for whom subject cards were issued (the rest did not visit the testing site or did not provide consent). Blood samples for haemoglobin measurements were obtained from 62 % of registered subjects (some tests were missed due to shortage of equipment).

### Malaria prevalence

**Fig. 1:**
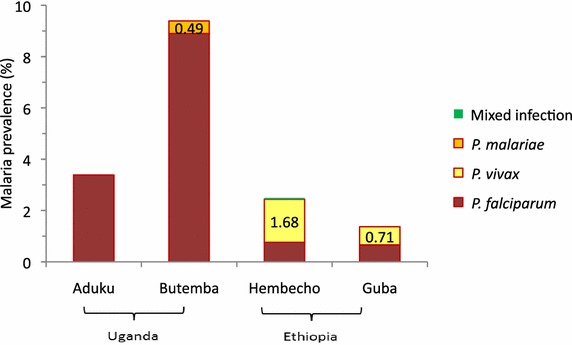
Malaria prevalence and composition of *Plasmodium* species in the study sites, October–November 2012

**Fig. 2:**
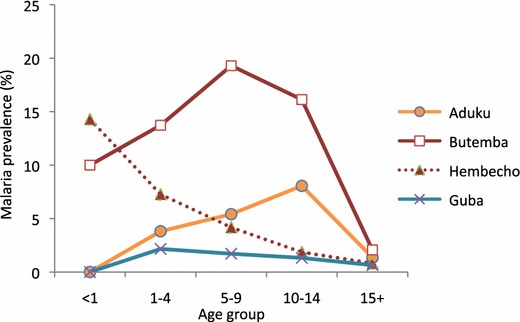
Malaria prevalence by age group (all species)

Prevalence of malaria infection varied between the four sites, ranging from 1.4 % in Guba to 9.9 % in Butemba (Fig. 1). The predominant species in Ethiopia was *Plasmodium vivax* which accounted for 52–67 % of all infections. In Uganda, *P. falciparum* accounted for 95–100 %. Malaria prevalence varied among age groups in most sites (Fig. 2). The risk of infection tended to be high in older subjects in Aduku. The age group pattern in both sites of Ethiopia was typical of low endemicity where all age groups are more-or-less equally affected, whereas the pattern in Butemba (Uganda) reflected moderate endemicity where malaria prevalence rates in older children and adults are less than rates in younger children as a result of acquisition of partial immunity due to repeated infections over time [39]. Aduku exhibited a pattern somewhat between low and moderate endemicity. The seemingly high prevalence in the under-one age group in the Hembecho site was due to a small sample size (one infection out of seven infants).

Subjects with fever had higher infection prevalence than those without fever in Butemba (*p* = 0.016) and Guba (*p* < 0.001). History of fever, however, had no association with malaria infection risk in all sites except in Guba where subjects with history of fever were more likely to be positive (X^2^ = 30.86, *p* < 0.001).

The positive and negative predictive values of RDTs for these cases were calculated for each site using the slide positivity rates determined by microscopy. The negative predictive value (NPV) of RDT for *P. falciparum* was high (≥99 %) in all three sites with sufficient sample sizes for this analysis (Aduku, Butemba and Hembecho). The positive predictive value (PPV) of RDT was low in all three sites (≤44 %). RDT results for *P. vivax* infection exhibited a similar pattern of high NPV (≥98 %) and low PPV (≤40 %) for the two sites in Ethiopia.

### Anaemia

There was a uniformly high prevalence of mild anaemia in all sites, ranging from 53 % in Aduku to 65 % in Hembecho. However, substantial variation was observed in prevalence of moderate and severe anaemia, ranging from 2.4 % in Aduku to 6.0 % in Butemba.

### Age-specific seroprevalence

**Fig. 3:**
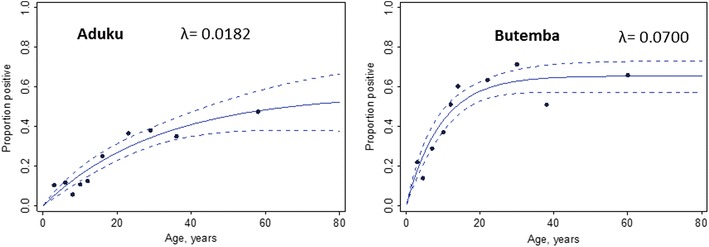
Age-specific seroprevalence for *P. falciparum* anti-MSP-1_19_ antibodies. *Dots*, *continuous lines* and *broken lines* represent data, fitted estimates and 95 % confidence intervals, respectively. λ is the seroconversion rate. The 0–2 years age group was omitted because of distortions caused by presence of maternal antibody in high endemicity settings [35]

A total of 1123 samples were analysed for the antigen MSP-1_19_, 602 for Aduku and 521 for Butemba. Overall seropositivity (i.e., the proportion of individuals with a positive antibody response to MSP-1_19_) was higher in Butemba (45.7 %) than Aduku (22.9 %), indicating higher overall exposure to malaria. The seroconversion rate (λ) was higher in Butemba compared to Aduku, indicative of a higher transmission intensity (Fig. 3).

### Under-five mortality rates

**Fig. 4:**
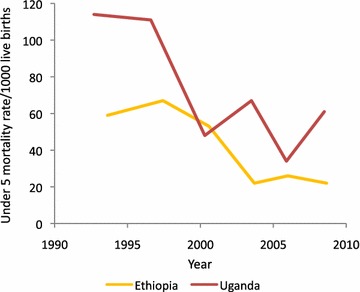
Under-5 mortality rate estimated from birth history data in Ethiopia and Uganda

Summary birth histories of 1503 women were available: 1041 women in Ethiopia and 462 in Uganda. Of these 88 % had complete data and were retained in the analysis. In total 3317 live births were reported in Ethiopia and 1101 in Uganda and the average number of live births to women aged 15–49 years was approximately 3.5 and 2.9 in Ethiopia and Uganda, respectively. As expected, the number of live births increased in older age groups. Mortality rates among live births were 4.1 % in Ethiopia and 6.2 % in Uganda. The trend observed for Ethiopia is a reduction of almost 60 % in the U5MR from approximately 60 deaths per 1000 live births for the years prior to 2000 to approximately 23 deaths per 1000 live births post-2000 (Fig. 4). A similar reduction was observed in Uganda. Pre-2000, the U5MR was approximately 113 per 1000 live births, whereas post-2000, the rates fluctuated between 34 and 67.

### Ownership and use of ITNs

ITN coverage varied between countries and sites. Hembecho had the highest coverage whereas Guba had the lowest (Table 2). Net ownership did not vary with SES. Purchasing of nets was evident in Uganda but not in Ethiopia. In Uganda, 42/606 (7 %) of the mosquito nets were purchased by the households themselves. None of these nets were identified as ITNs (15 were not ITNs and 27 were of unknown type). Thirty-one were bought from open market, 10 from shops and 1 from a pharmacy. The proportion of nets purchased was not higher in the upper (richer) socioeconomic groups.

**Table 2:** ITN ownership and use rates

Indicator	Uganda		Ethiopia	
	Aduku	Butemba	Hembecho	Guba
% households with at least 1 ITN for 2 people	24.3	30.3	24.1	3.6
% households with at least 1 ITN	77.5	67.8	98.2	55.8
% households with at least 2 ITNs	19.5	42.2	73.3	9.8
Number of ITNs per household	1.1	1.5	1.9	0.7
Number of residents per household	5.0	5.3	5.2	5.2
Number of sleeping places	2.5	2.1	2.1	1.9
Number of persons per sleeping place	2.0	2.5	2.5	2.7
% of ITNs used the previous night	81.8	80.0	78.9	28.6
% of people with access to ITNs	43.0	49.2	67.6	25.0
% of people with access who used ITNs the previous night	74.9	69.4	76.1	33.4

Access of household members to ITNs was calculated for each study site. To assess access of nets within the household, first the potential number of people who could use the available nets was computed assuming one net for two people. When the potential number of people who could use the available nets exceeded the total number of people who spent the previous night in the household, the two figures were assumed to be equal. The sum of all potential users in the sample was then divided by the total number of people who spent the previous night in surveyed households to estimate the access rates or the proportion of population with access to an ITN within the household.

The ITN use rates among those who have access were then estimated for each site. The data show that there was variation between and within countries in terms of access to nets. Hembecho had the highest access rate while Guba had the lowest.

Among people with access to ITNs, use rates were high in three of the four sites (ranging from 69 to 76 %), with the exception of Guba in Ethiopia, where only a third of those with access used a ITN the previous night. There was no inequity between the sexes in ITN use for any site, except for adults in Aduku, where significantly more females used an ITN (OR = 1.6; 95 % CI 1.1–2.2).

### Indoor residual spraying coverage

No IRS was carried out in Butemba. The remaining three study areas contained households that had been sprayed in the last 12 months, with or without concurrent ITN use. The coverage of IRS was consistently high across the villages within each study site. On average, 84 % of households in Aduku were sprayed within the last 12 months, 96 % in Hembecho and 85 % in Guba.

### Prevalence of infection by use of preventive measures

Individuals who used an ITN the night before the survey had significantly lower malaria infection prevalence in Aduku (2.3 % versus 5.7 %; *p* = 0.020). No statistically significant difference was observed in the other sites. There was no statistically significant difference in malaria infection prevalence between houses with open/partially open or closed eaves in all sites. However, in Guba (the site with the lowest ITN ownership and use rates), houses with open eaves had slightly higher risk of infection with *P. falciparum* although this was not statistically significant (*p* = 0.087).

Out of the three sites where IRS was implemented, prevalence of infection was significantly lower in individuals living in sprayed houses compared to those in unsprayed houses only in Guba (*p* = 0.042). Among individuals who slept in sprayed houses, the prevalence of infection was significantly lower in those who used ITNs in Aduku (*p* = 0.026), indicating that ITNs are more protective in this site than they are in the other sites. No such association was observed in the other two sprayed sites.

### Febrile illness in children and treatment-seeking behaviour

A higher proportion of children under 10 years of age had fever or history of fever in the two Ugandan sites compared to the Ethiopian sites (Table 3). In Uganda, treatment was sought for more than 90 % of all children with fever, whereas in Ethiopia, this ranged between 62 and 80 %. In Aduku, first treatment was most commonly sought from privately owned clinics, pharmacies or drug shops, while in Butemba similar proportions were treated in the public and private sectors. In both Ethiopian sites, first treatment was primarily sought in the public sector. Village Health Teams (VHTs) in Uganda and Health Extension Workers (HEWs) in Ethiopia played important roles in providing early diagnosis and treatment of uncomplicated malaria within their communities.

**Table 3:** Prevalence and treatment of fevers in children (10 years or younger) by study site

Site	Aduku	Butemba	Hembecho	Guba
Number of children with fever	35	59	42	76
% children with fever in last 48 h	10.3	15.8	4.4	7.3
% children with fever (or history of fever) for whom treatment was sought	94.3	91.5	61.9	80.3
% febrile children who sought treatment in the public sector among those who sought treatment	33.3	48.0	72.2	91.8
% febrile children given any antimalarial (% ACT use)	77.1 (55.6)	72.9 (79.1)	35.7 (86.7)	73.7 (41.1)
% febrile children treated with antimalarial within 24 h of onset of fever	44.4	53.5	6.7	1.8
% of febrile children for whom treatment was sought from any source who were tested	51.6	60.4	76.9	91.8
% of the tests that were done in public health facilities	62.5	75.0	100.0	96.4

Anti-malarials were given to the majority of children with fevers in all sites with the exception of Hembecho, where only 36 % of children received an anti-malarial. ACT use varied across the sites (In Ethiopia, chloroquine was used as well as ACT as it was first-line treatment for vivax malaria). For approximately half of the children that were given an anti-malarial in Uganda, treatment was started within 24 h following onset of fever. In Ethiopia, only 2–7 % of children who received an anti-malarial started the treatment within 24 h of onset of fever. Approximately 52–60 % and 77–92 % of children with fever who sought treatment received a malaria diagnostic test (either RDT or microscopy) in Uganda and Ethiopia, respectively.

### Prevention of malaria in pregnancy

Overall, IPTp use was more common in Butemba compared to Aduku (X^2^ = 15.1, *p* = 0.004), and 36.9 % of the women who gave birth within 2 years preceding the survey took two doses of IPTp, compared to 20.5 % in Aduku (Table 4). The proportion of women that took three or more doses was around 7–8 % in both sites (IPTp is not implemented in Ethiopia).

**Table 4:** IPTp use by women who gave birth in the previous 2 years before the survey, Uganda

Site	n	IPTp (%)				
		None	1 Dose	2 Doses	3 Doses	Don’t know
Aduku	39	53.8	10.3	20.5	7.7	7.7
Butemba	84	20.2	25.0	36.9	7.1	10.7

IPTp is not part of the national malaria control strategy in Ethiopia

### Knowledge about malaria

A large majority of respondents in Uganda identified mosquito bites as the cause of malaria (93 % in Aduku and 77 % in Butemba), whereas in Ethiopia the percentages were relatively lower (65 % in Hembecho and 39 % in Guba). In Uganda, 89 and 79 % of respondents had heard information about malaria in Aduku and Butemba, respectively, whereas messages about malaria were less frequently heard in Ethiopia (X^*2*^ = 158.6, *p* < 0.001) (61 % in Hembecho and 36 % in Guba). The main sources of information in the Uganda sites were radio and health workers, while in Ethiopia, community leaders and health workers were mentioned most often.

### Mosquito density and species composition

In Ethiopia, only 22 *A. gambiae* s.l. females were collected from both sites, which were probably *A. arabiensis*. In Uganda, a total of 1670 and 315 anopheline females were collected using the various trapping techniques in the Aduku and Butemba sites, respectively. In Aduku, the most common vector species was *A. funestus* s.l. (53 % of the collections), while *A. gambiae* s.l. made up only 18 % of the collections. The majority of other anophelines considered as non-vectors belonged to the *Anopheles coustani* complex. In Aduku, 94 % of the *A. gambiae* s.l. were identified as *A. arabiensis* and 6 % as *A. gambiae* s.s. (molecular identification of *A. funestus* s.l. is yet to be completed).

In Butemba, *A. gambiae* s.l. was the dominant species (89 % of all collections) and no *A. funestus* s.l. was collected. Molecular analysis showed that 93 % of all *A. gambiae* s.l. mosquitoes collected in Butemba were *A. gambiae* s.s. while 7 % were *A. arabiensis*.

### Vector biting habits

**Fig. 5:**
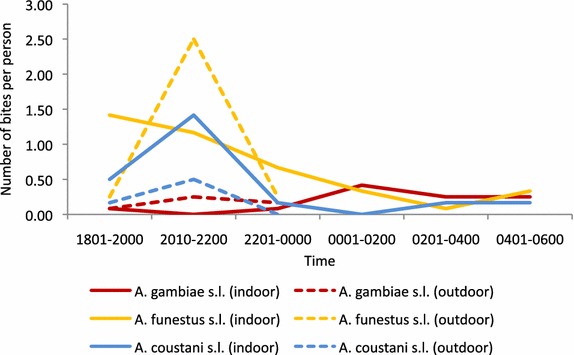
Nocturnal biting cycle of *Anopheles* species in Aduku, Apac District. Collection hours: indoors 1801–0600 h and outdoors 1801–0000 h

In Butemba, the only vector species caught by HLC was *A. gambiae* s.l. However, only seven mosquitoes were caught indoors which was not sufficient to deduce any feeding patterns. In Aduku, the *A. gambiae* s.l. identified were 17 *A. arabiensis* and one *A. gambiae* s.s. These were observed to feed primarily after midnight indoors (Fig. 5). The number collected was too few to determine any behavioural differences between the sibling species. *A. funestus* s.l. was observed to feed much earlier in the evening both indoors and outdoors. This result, however, requires confirmation and as the molecular analysis of *A. funestus* s.l. has not been completed to confirm if the species under question is an important vector, this result should be interpreted with caution. Additionally, *A. coustani* s.l. showed a similar feeding pattern to *A. funestus* s.l.

Human biting rates (HBR) for all anopheline species combined in Aduku was 7.3 anopheline bites per person night (Table 5). *Anopheles funestus* s.l. was the largest contributor to the HBR followed by *A. coustani* s.l. and *A. gambiae* s.l. In Butemba, the HBR was 0.5 bites per person night. If given equal opportunity (or if human baits are present both indoors and outdoors) during 1800–0000 h, *A. gambiae* s.l. showed a clear preference for biting outdoors (75 %) in Aduku. In contrast, *A. coustani* s.l. clearly preferred biting indoors (only 24 % preferred biting outdoors), whereas *A. funestus* s.l. readily fed both indoors and outdoors.

**Table 5:** Human biting rate (HBR) for each species in study sites in Uganda

Site	Details	*A. gambiae* s.l.	*A. funestus* s.l.	*A. coustani* s.l.
Aduku	Sample size (indoors + outdoors)	19	84	37
	Total HBR per person per night^a^	1.15	4.04	2.10
	% human-vector contact occurring indoors	92.7	83.0	92.1
	% feeding on humans before 2200 h (indoors + outdoors)	12.7	64.9	76.2
	% feeding outdoors if given equal opportunity (exophagy)^b^	75.0	48.0	24.2
Butemba	Sample size (indoors and outdoors)	7	0	0
	Total HBR per person per night	0.52	0	0

^a^HBR is expressed as the average number of bites per person per night. It was assumed that an average villager spends 1 h outdoors between 1800 and 2200 h and the remainder of the night indoors

^b^Only collections between 1800 and 0000 h were used for both indoors and outdoors for calculations of the extent of exophagic habit of the vector independent of night time habits of humans

Nevertheless, assuming that an average villager spends one hour outside prior to 2200 h and is indoors after 2200 h, the majority of human-vector contact for all three species occurred indoors, ranging from 83 % for *A. funestus* s.l. to 93 % for *A. gambiae* s.l. Assuming that by 2200 h all residents would go to bed, the proportion of contact before this time could be used as a proxy for the potential risk of malaria exposure. The largest proportion of human-vector contact for *A. funestus* s.l. (65 %) and *A. coustani* s.l. (76 %) took place before 2200 h. For *A. gambiae* s.l. only 13 % of the human-vector contact occurred before 2200 h. As mentioned above the results for *A. funestus* s.l. should be interpreted fully only when species data is available.

### *Kdr*-L1014S genotype frequencies

In Butemba, *kdr*-L1014S (*kdr*-*east*) frequencies were 94.7 % (*n* = 113) in *A. gambiae* s.s.; no susceptible individuals were observed. The few *A. arabiensis* analysed (*n* = 7) were all susceptible. In Aduku, *A. gambiae* s.s. showed a high L1014S frequency of 81 % (*n* = 13), while for *A. arabiensis* frequency was 3 % (*n* = 252).

### Undernutrition

**Fig. 6:**
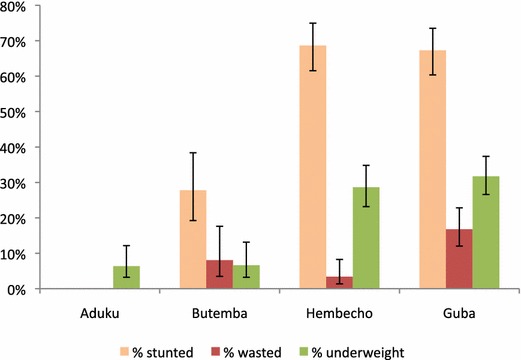
Percentage of stunted, wasted and underweight children under five Uganda and Ethiopia. *Error bars* indicate 95 % confidence intervals. Height measurements were not taken at Aduku resulting in absence of stunting and wasting data

Undernutrition was an important problem in both countries. However, a far greater proportion of children under five in the Ethiopian sites (29-32 %) were underweight compared with the Ugandan sites (6–7 %). The Ethiopia sites had the highest percentage of children affected by undernutrition. Approximately two-thirds of children in the Ethiopian sites were stunted (Fig. 6). Wasting, which is an indicator of acute undernutrition, was most prevalent in the Guba site (16.8 %) and affected a much lower proportion of children in the Hembecho site (3.4 %). In the Butemba site of Uganda, more than a quarter of children under five were stunted (no data was collected on stunting and wasting in Aduku). In Butemba, 8.1 % of children were affected.

## Discussion

A comprehensive survey was carried out in four sites in Ethiopia and Uganda to gather baseline data for future surveys aimed at monitoring changes in malaria epidemiology and effectiveness of interventions over time. Malaria transmission during October–November 2012 in all locations was low or moderate including in previously highly endemic areas although the surveys were conducted during months normally considered as falling in the peak transmission season.

Malaria prevalence in the Ugandan sites was much lower than historically recorded, particularly in the district of Apac [40]. However, as the studies are only in few sites, they may not reflect national trends. It is likely that the combined use of both IRS and LLINs led to the observed decline. Apac has been under the IRS programme since 2007. As baseline transmission levels vary across sites, it is inappropriate to compare the four study sites; however, it is worth noting that the highest malaria prevalence was observed in the site that was not under IRS in Uganda. The point prevalence data may not represent the actual average situation in a site. There is considerable seasonality of transmission in both countries as well as inter-annual variations. Especially in Ethiopia, the variations are likely to be governed by meteorological conditions [41, 42]. It is not always possible to accurately determine the peak of the transmission seasons correctly. In Ethiopia, prevalence rates were generally comparable to rates reported in the 2011 MIS survey [14]. However, the MIS survey reported dominance of *P. falciparum* contrary to the finding of the present study the previous year. The dominance of *P. vivax* in the present study could be due to reduced transmission conditions either as the result of transmission factors being relatively less favourable for *P. falciparum* or overall reduction of transmission due to the effects of interventions. In Uganda, however, prevalence rates were much lower than rates reported in the 2009 MIS survey during the same season [19].

Serology data can indicate systematic changes in transmission intensity by looking at the age-seroprevalence distribution [35]. Serological data confirm a higher level of transmission in Butemba compared to Aduku in Uganda. The age group pattern of infection prevalence also indicates a relatively high transmission situation in Butemba. The model does not fit some of the younger age groups well in Aduku, which could reflect transmission changes due to the ongoing IRS programme using the carbamate insecticide bendiocarb. Similarly, in Butemba, the youngest age group shows a lower than expected seroprevalence rate, which could be due to the LLIN mass distribution campaign in 2010. However, small sample sizes in younger age groups could also account for these findings, and additional serology data from subsequent rounds and additional analyses of samples from Ethiopia, as well as use of other antigens, can provide more robust datasets to analyse the interventions in terms of transmission changes.

Entomological results yielded important information on the situation of vectors. *A. gambiae* s.s. dominated in Butemba, while in Aduku, *A. funestus* s.l. and *A. arabiensis* were the main vectors. Prior to the scale up of interventions, *A. funestus* s.l. was the main vector in Apac [40], suggesting more success of the vector control efforts against this vector. Most human-vector contact with *A. gambiae* s.l. occurs indoors in Aduku, Uganda, which shows that the use of interventions against indoor-biting vectors should be considered effective. However, the early feeding observed for *A. funestus* s.l. requires confirmation with data from subsequent surveys. A substantial number of *A. coustani* s.l. were collected by human landing catch suggesting attraction to humans. Its status as a malaria vector is unknown in Uganda. A study in Zambia found a high degree of anthropophily in *A. coustani* s.l. [43] and it was reported to be contributing to transmission in Kenya [44]. Forthcoming analyses will determine infectivity rates of vectors to calculate entomological inoculation rates.

Insecticide resistance is increasing in many areas of Uganda [45, 46]. High *kdr*-L1014S frequencies were observed in *A. gambiae* s.s. and low frequencies in *A. arabiensis*, a finding which was in line with another study from Eastern Uganda [47]. Further studies will be needed to confirm the magnitude and impact of insecticide resistance in both countries. No tests were done in Ethiopia due to small sample size but widespread resistance to DDT and pyrethroids has been reported [16, 48]. Resistance against pyrethroids and DDT has reached a high level in both countries, probably due to the substantial increase in the distribution and use of LLINs in recent years as these insecticides share a similar mode of action in terms of knock-down resistance.

Regarding diagnosis and treatment, there are variations observed in both countries, especially around use of services. In Uganda, seeking treatment in the private sector was common. These findings are in line with the MIS 2009 survey, which observed that treatment was sought for 82 % of fevers of children under five treatment and 56 % of children with a fever were taken to the private sector [19, 49]. In Ethiopia, a great majority of febrile children were taken to public health facilities for treatment.

As part of the malariometric survey, febrile subjects were tested with RDTs which provided an opportunity to compare with subsequent microscopy results. The RDTs used had high negative predictive value, but their positive predictive value was uniformly low in all sites. This shows that negative results with the RDTs for febrile patients can be a good indicator of absence of infection but a positive result may not always be a reliable indicator of presence of infection. These results may be explained by the nature of the RDTs that test for the presence of parasite antigens which circulate in the blood for several weeks post-infection [50, 51].

Prevention of malaria in pregnancy was one of the interventions implemented in Uganda. The survey results indicated that IPTp uptake was low in Uganda and needs to be strengthened if the current recommendation of at least three doses is to be reached [52]. The rather low coverage or use levels are in line with findings from the DHS survey in 2011 which found that 25 % of women reported to have taken at least two doses of IPTp [23]. While data for Aduku was similar to the national average in 2011, in Butemba a higher proportion of women reported using IPTp, likely due to activities of various projects in this region working to improve its uptake.

Knowledge of malaria varied among the sites. Households in the Ugandan sites had overall better knowledge about malaria compared to the Ethiopian sites. However, in Butemba, other causes of malaria were frequently cited, unlike in Aduku where most respondents correctly identified mosquitoes as the only cause of malaria, likely because of behavioural change communication (BCC) campaigns that accompanied IRS implementation. In Guba, knowledge about the cause of malaria needs to be improved through appropriate community education. In connection with this, the low ITN ownership and use rates in Guba and possibly surrounding areas in Ethiopia will require close attention by the health services.

Universal coverage is defined differently in the two countries: two ITNs per household in Ethiopia and one ITN for two people in Uganda. In one of the Ethiopian sites (Hembecho), the goal was nearly achieved with 1.9 ITNs per household whereas ownership in the second site (Guba) was the lowest of all sites (0.7 ITNs). The cause of the low ownership rate in Guba requires more investigation. In Uganda, although the percentages of sleeping places that can potentially be covered with ITNs available in the households were 44 and 71 % in Aduku and Butemba, respectively, the percentages of households with one ITN for two people were 24 and 30 %, respectively. The ITN coverage is expected to increase substantially following a recent nationwide campaign in Uganda.

Uniformly high IRS coverage was observed in the sprayed sites. In one of the four sites (Aduku), individuals who slept under an ITN the night before the survey had lower risk of infection than those who did not. This could be due to low level of pyrethroid resistance in that site [53]. The lack of association in ITN use and malaria in the other sites does not necessarily translate to lack of effectiveness. It may be partly explained by ‘mass’ effects of ITNs or IRS (in the sprayed sites) which could confound the result, as people who did not use nets might have been ‘protected’ by either all other nets in the villages or the IRS due to mortality effects on mosquito vectors or a combination of these.

It is not clear whether ITN and IRS have an additive or synergistic action when used in combination. The current WHO recommendation is that where LLIN coverage is high and they remain effective, IRS may have limited utility in reducing malaria morbidity and mortality, unless the combined use is for resistance management [54]. In Guba where both ITN ownership and use rates were low, malaria infection risk was higher in individuals living in unsprayed houses than in sprayed ones, indicating the potential benefit of the combined use of both interventions in such situations.

One of the main observations in the present survey was the high level of undernutrition in the study sites. Undernutrition during the critical first 1000 days of a child’s life could have devastating consequences by increasing morbidity and mortality and development of the child. Recently, stunting (or low height for age) has been chosen as a key indicator to measure global and national progress towards reduction of undernutrition [55]. Undernutrition is particularly a severe problem in the Ethiopia sites where two-thirds of the children were classified as stunted. Critical nutrition interventions should be strengthened addressing both maternal and child undernutrition. These include, among others, promoting optimal breastfeeding practices, micronutrient supplementation, reducing incidence of low birth weight and prevention of disease.

## Conclusion

Low malaria prevalence was observed in some sites that previously had high endemicity but there was substantial variation between sites. Intensified vector control and effective treatment seem to have played key roles in bringing endemicity down over recent years, as reflected in the results of serological analyses. Malaria control efforts should be sustained to reduce transmission further, maintain the gains and prevent resurgence. Control strategies should be adapted to the changing patterns and heterogeneity of transmission which may require a thorough epidemiological stratification and selective targeting of interventions. A tendency of early biting in *A. funestus* s.l. in Aduku requires further investigation. The impact of pyrethroid resistance in *A. gambiae* s.s. in Uganda on effectiveness of LLINs should be studied further. Pre-emptive rotation of insecticides should be considered by IRS programmes. Non-pyrethroid IRS may be considered when feasible and where other measures have inadequate impact, or in areas where there are major obstacles to achieving high ownership and use of LLINs. Robust continuous distribution systems are needed to replace LLINs and maintain high coverage. Whether low LLIN coverage in Guba is indicating a similar problem elsewhere should be investigated and more efforts are needed to create a culture of net use. Health services should create conditions to make LLINs available in the commercial market at affordable prices and educate communities on the benefits of LLINs over untreated nets.

The findings will serve as a baseline against which results of future surveys or other studies could be compared. Although results from a small number of study sites may not be nationally representative, the approach provides more comprehensive information on a range of potential determinants of malaria rates than more geographically extensive surveys and surveillance, and they will provide a basis for and may prompt further investigations of some of the observations. It is necessary to continue monitoring the epidemiological changes and more studies through in-depth analysis and modelling will reveal the impact of the various interventions on malaria transmission.
