# Correction to: Monitoring changes in malaria epidemiology and effectiveness of interventions in Ethiopia and Uganda: Beyond Garki Project baseline survey

**DOI:** 10.1186/s12936-019-3006-5

**Published:** 2019-11-25

**Authors:** Tarekegn A. Abeku, Michelle E. H. Helinski, Matthew J. Kirby, Takele Kefyalew, Tessema Awano, Esey Batisso, Gezahegn Tesfaye, James Ssekitooleko, Sarala Nicholas, Laura Erdmanis, Angela Nalwoga, Chris Bass, Stephen Cose, Ashenafi Assefa, Zelalem Kebede, Tedila Habte, Vincent Katamba, Anthony Nuwa, Stella Bakeera-Ssali, Sarah C. Akiror, Irene Kyomuhangi, Agonafer Tekalegne, Godfrey Magumba, Sylvia R. Meek

**Affiliations:** 1 Malaria Consortium, London, UK; 2 Malaria Consortium, Addis Ababa, Ethiopia; 3 Malaria Consortium, Kampala, Uganda; 4 Rothamsted Research, Harpenden, UK; 5 Medical Research Council/Uganda Virus Research Institute, Uganda Research Unit on AIDS, Entebbe, Uganda; 6 South Nations, Nationalities and Peoples Regional Health Bureaux, Hawassa, Ethiopia; 7 London School of Hygiene & Tropical Medicine, London, UK; 8 Ethiopian Public Health Institute, Addis Ababa, Ethiopia; 9 National Malaria Control Programme, Ministry of Health, Kampala, Uganda

## Correction to: Malar J (2015) 14:337 10.1186/s12936-015-0852-7

Please be advised that one of the author names is incorrectly spelled in the published article: ‘Irene Kyomuhagi’ should be ‘Irene Kyomuhangi’.

The corrected name can be found in the author list of this article.

## References

[1] Abeku TA, Helinski MEH, Kirby MJ, Kefyalew T, Awano T, Batisso E, Tesfaye G, Ssekitooleko J, Nicholas S, Erdmanis L, Nalwoga A, Bass C, Cose S, Assefa A, Kebede Z, Habte T, Katamba V, Nuwa A, Bakeera-Ssali S, Akiror SC, Kyomuhangi I, Tekalegne A, Magumba G, Meek SR (2015). Monitoring changes in malaria epidemiology and effectiveness of interventions in Ethiopia and Uganda: beyond Garki Project baseline survey. Malar J.

